# Comparison of concurrent validity of different malnutrition screening tools with the Global Leadership Initiative on Malnutrition (GLIM) among stroke survivors in Malaysia

**DOI:** 10.1038/s41598-023-31006-y

**Published:** 2023-03-30

**Authors:** Hui Jie Wong, Sakinah Harith, Pei Lin Lua, Khairul Azmi Ibrahim

**Affiliations:** 1 Faculty of Health Sciences, School of Nutrition and Dietetics, Universiti Sultan Zainal Abidin, Gong Badak Campus, 21300 Kuala Nerus, Terengganu Malaysia; 2 Faculty of Health Sciences, Universiti Sultan Zainal Abidin, Gong Badak Campus, 21300 Kuala Nerus, Terengganu Malaysia; 3 Faculty of Pharmacy, Universiti Sultan Zainal Abidin, Besut Campus, 22200 Besut, Terengganu Malaysia; 4 Neurology Unit, Department of Medicine, Hospital Sultanah Nur Zahirah, Ministry of Health Malaysia, Jalan Sultan Mahmud, 20400 Kuala Nerus, Terengganu Malaysia; 5 Dietetic Unit, Karak Health Clinic, Ministry of Health Malaysia, Jalan Besar Karak, 28600 Bentong, Pahang Malaysia

**Keywords:** Malnutrition, Stroke

## Abstract

Individuals with stroke are at high malnutrition risk in both the acute and chronic phases. This study aimed to assess the validity of different malnutrition screening tools for stroke patients in rehabilitation phase. Participants in this study were 304 stroke patients from three hospitals in the East-Coast region of Peninsular Malaysia from May–August 2019. The concurrent validity of the Malnutrition Risk Screening Tool-Hospital (MRST-H), Mini Nutritional Assessment-Short Form (MNA-SF), Malnutrition Screening Tool (MST), Malnutrition Universal Screening (MUST) and Nutritional Risk Screening (NRS-2002) was assessed with the diagnostic criteria for malnutrition proposed by the Global Leadership Initiative on Malnutrition (GLIM-DCM). Sensitivity, specificity, positive predictive value, negative predictive value, and the area under the curve were computed. MUST and MRST-H demonstrated good validity regardless of different age groups (> 80% sensitivity and specificity); meanwhile, MST and MNA-SF had fair validity, yet NRS-2002 had poor to fair validity with GLIM-DCM. Only MRST-H and NRS-2002 were significantly correlated with all anthropometric indices, dietary energy intake, and health-related quality of life in both age groups. In conclusion, MRST-H and MUST showed good concurrent validity with GLIM-DCM and can be considered as appropriate malnutrition screening tool in discriminating malnutrition among stroke individuals attending rehabilitation centre in Malaysia regardless of their age groups.

## Introduction

Individuals with stroke are subjected to high malnutrition risk, with prevalence at 46–91% in the rehabilitation phase^1–4^. The differences in the prevalence could be attributed to methodological and respondent characteristics differences between the studies (e.g., elderly versus non-elderly, subacute versus chronic phase, different nutritional screening instruments, and severity of stroke)^5^. There is no gold standard to assess malnutrition. Malnutrition has been defined based on a single or combination of nutritional parameters such as body mass index (BMI), calf circumference (CC), mid-upper arm circumference (MUAC), triceps skinfold, changes in body weight, or clinical laboratory parameters (e.g. serum albumin levels and lymphocyte count)^5^. The European Society of Clinical Nutrition and Metabolism (ESPEN) and the American Society of Parenteral and Enteral Nutrition (ASPEN) and the Academy of Nutrition and Dietetics had published definitions of malnutrition, although discrepancies between these definitions are apparent^6,7^. Other experts however have suggested clinical assessment by a nutritionally trained professional (e.g. dietitian), or using the Subjective Global Assessment (SGA) for all adults, and the full form of Mini Nutritional Assessment (MNA) for elderly as the semi-gold reference standards^8^.

Recently, a core leadership committee with representatives of several of the global clinical nutrition societies [ESPEN, ASPEN and the Academy of Nutrition and Dietetics, PELANPE, and PENSA] have come into a consensus on the diagnostic criteria for malnutrition (DCM)^9^. The Global Leadership Initiative on Malnutrition (GLIM) proposed a two steps approach encompasses of malnutrition risk screening (step 1) followed by assessment for diagnosis (step 2). The GLIM suggested the use of any validated screening tools in the step 1 process^9^. These included the Mini Nutritional Assessment-Short Form (MNA-SF), Malnutrition Screening Tool (MST), Malnutrition Universal Screening (MUST), and Nutritional Risk Screening (NRS 2002).

However, most of these screening tools were developed in western countries, and their validity among the Asian population is questionable and warrants further investigation^8^. There are screening tools developed and validated specifically for the local population in Asian countries, namely Chinese Nutrition Screen (CNS) (China), Malnutrition Risk Screening Tool-Hospital (Malaysia), Malnutrition Risk Screening Tool-Community (Malaysia), and the 3-Minute Nutrition Screening (3-MinNS) (Singapore)^10–13^. Although different screening tools are in use, their levels of validity, agreement, reliability, and generalizability vary^14^. Additionally, age-, disease-, or setting-specific screening tools exist (hospital, community, or residential care), although these are subjected to practical limitations. Some tools such as MUST and MST have been widely used for adults and validated in acute and community settings^8^. Meanwhile, MRST-H and MNA-SF were specifically designed for the elderly population in acute and community settings^8,15^. In contrast, NRS-2002 was developed mainly for adults in the acute setting^16^. There is however no preferred malnutrition screening tool to screen malnutrition risk for stroke individuals in rehabilitation phase. Evidence on validity of malnutrition screening tools in the rehabilitation setting is limited^8,17^.

Previous studies have compared the criterion validity of these malnutrition screening tools with different reference standards proposed by different expert groups, namely clinical assessment by dietitian, SGA, MNA- long forms, ESPEN, and ASPEN^8^. To our best knowledge, no studies have compared the criterion validity of these different malnutrition screening tools with the diagnostic criteria proposed by GLIM. It is important to validate these screening tools with the newly proposed GLIM diagnostic criteria since it will affect the number of participants being assessed in the following step. For example, some individuals who were screened as not having a malnutrition risk by the MST would have been identified as malnourished on the basis of a BMI < 18.5 kg/m^2^. In order to improve the nutritional status of patients with high risk of malnutrition, clinicians need to know the most valid screening tools for identifying malnutrition risk in Malaysia context to allow further assessment and early treatment. Therefore, the main objective of this study is to compare the concurrent validity of different malnutrition risk screening tools with the GLIM-DCM among stroke survivors. The validity of these screening tools was also compared among elderly and non-elderly groups.

## Methods

### Study design and setting

Respondents in this cross-sectional study were individuals with stroke recruited from three general public hospitals in the East-Coast region of Peninsular Malaysia from May to August 2019. During their waiting hours at the neurology and rehabilitation departments, these respondents were invited.

### Participants

Participants who were more than 18 years old and had received a stroke diagnosis as confirmed by a medical doctor (ICD 160–169) were eligible to participate in the study. Participants were excluded if they were suffering from hemiparesis or contracture deformity that affected anthropometric assessment. All stroke participants who have attended the Neurology and Rehabilitation departments in the three selected hospitals during the study period were approached and screened based on inclusion and exclusion criteria. All participants who agreed to join the study were recruited. All eligible participants were assessed and interviewed face-to-face by a clinically trained dietitian.

### Sample size

We calculated the sample size using the formula below, where Z_α_ is the standard normal deviate, P is the expected sensitivity of the screening tool, and W is the width of the confidence interval (CI):

$${\text{Sample}}\;{\text{size}}\;({\text{N}}) = \frac{{4Z_{\alpha }^{2} P(1 - P)}}{{W^{2} }}$$

Marshall et al. reported that the sensitivity of MST in detecting malnutrition in geriatric rehabilitation was 80.8%. By referring to Marshall et al.'s finding, a standard normal deviate at 1.96 (with a 95% CI), a width of the CI being ± 0.10, and a 20% non-response rate, the required sample size was 300^17^.

### Sociodemographic and clinical characteristics

The following sociodemographic and clinical profiles were collected during the survey: sex, ethnicity, age, educational level, household income, underlying comorbidities, duration of a stroke, types of stroke and episodes of stroke. Besides, participants were also asked whether they always faced problems (Yes or No) such as nausea, vomiting, chewing, swallowing, loss of appetite, speech, memory, constipation, diarrhoea, and paresis of the dominant arm.

### Malnutrition risk screening tools

The risk of malnutrition was assessed by the MRST-H, MNA-SF, MST, MUST, and NRS-2002. Screening criteria and scores in each screening tool are displayed in Table 1. Some questions are similar across different tools, including BMI, weight loss and decreased foods intake. Thus, these questions were only asked once to prevent unnecessary burden on the participants. A participant was classified as having malnutrition risk if obtained scores ≥ 2 for MRST-H, ≥ 1 for MUST, ≥ 2 for MST, ≤ 11 for MNA-SF and ≥ 3 for NRS-2002^15,16,18–20^.

**Table 1:** Screening criteria for different malnutrition screening tools. *Source*: Tan et al.^15^, Ferguson et al.^18^, Todorovic and Elia^19^, Rubenstein et al.^20^, Kondrup et al.^16^.

Criteria	MRST-H	MUST	MST	MNA-SF	NRS-2002
BMI (kg/m^2^)	–	0 = > 20 1 = 18.5–20 2 = < 18.5	–	0 = < 19 1 = 19–20.9 2 = 21–22.9 3 = ≥ 23	Impaired nutritional status: 0 = Normal 1 = Weight loss > 5% in 3 months or Food intake 50–75% of normal requirement in preceding week 2 = Weight loss > 5% in 2 months or BMI 18.5 – 20.5 + impaired general condition or Food intake 25–60% 3 = Weight loss > 5% in 1 month (> 15% in 3 months) or BMI < 18.5 + impaired general condition or Food intake 0–25% Impaired general condition (e.g. weak but out of bed, confined to bed, or in intensive care)
Unplanned weight loss	3 = > 5% 1 month or > 10% 6 months 0 = No weight loss	For past 3–6 months: 0 = < 5% 1 = 5–10% 2 = > 10%	For the past 6 months: 0 = No 2 = Unsure 1 = 1–5 kg 2 = 6–10 kg 3 = 11–15 kg 4 = > 15 kg	For the past 3 months: 0 = > 3 kg 1 = Does not know 2 = 1–3 kg 3 = No weight loss	
Food intake	Unable to feed or eat by own self 0 = No 1 = Yes	–	Eating poorly (a decreased appetite) 0 = No 1 = Yes	0 = Severe decrease 1 = Moderate decrease 2 = No decrease	
Mobility	–	–		0 = Bed or chair bound 1 = Able to get out of bed/chair, but does not go out 2 = Goes out	
Acute disease effect	–	2 = Acutely ill and there has been or is likely to be no nutritional intake for > 5 days 0 = Not acutely ill	–	Psychological stress or acute disease in the past three months? 0 = Yes 2 = No	Severity of disease: 0 = Normal requirements 1 = Hip fracture, chronic patients, cirrhosis, COPD, hemodialysis, diabetes, oncology 2 = Major abdominal surgery, severe pneumonia, hematologic malignancy 3 = Head injury, bone marrow transplantation, intensive care
Depression/ Dementia	–	–	–	0 = Severe 1 = Mild 2 = No problems	–
MUAC (cm)	0 = ≥ 23.0 (male); 22.0 (female) 2 = < 23.0 (male); 22.0 (female)	–	–	–	–
CC (cm)	0 = ≥ 30.1 (male); 27.3 (female) 1 = < 30.1 (male); 27.3 (female)	–	–	0 = < 31 3 = ≥ 31 (if BMI is not available)	–
Age factor	–	–	–	–	0 = < 70 years old 1 = ≥ 70 years old
Total scores	< 2 = Low risk ≥ 2 = High risk	0 = Low risk 1 = Medium risk ≥ 2 = High risk	0–1 = Low risk 2 = Moderate risk 3–5 = High risk	0–7 = Malnourished 8–11 = At risk of malnutrition 12–14 = Normal	Total scores = impaired nutritional status (0–3) + severity of disease (0–3) + age factor (0–1) ≥ 3 = Nutritionally at risk

*MRST-H* Malnutrition Risk Screening Tool-Hospital, *MNA-SF* Mini Nutritional Assessment-Short Form, *MST* Malnutrition Screening Tool, *MUST* Malnutrition Universal Screening, *NRS-2002* Nutritional Risk Screening, *MUAC* Mid-upper arm circumference, *CC* Calf circumference, *COPD* Chronic obstructive pulmonary disease.

This study used the malnutrition diagnosis criteria as proposed by the GLIM (Table 2)^9^. The GLIM proposed five criteria: non-volitional weight loss, low body mass index, reduced muscle mass, reduced food intake or assimilation, and disease burden/inflammation^9^. According to the GLIM-DCM, a participant is diagnosed with malnutrition if he or she fulfils at least one phenotypic criterion and one etiologic criterion. Since skeletal muscle index (SMI) was not being measured in this study, thus alternative indicators such as MUAC, CC and handgrip strength (HGS) were being used. Cut-off points for low muscle mass were proposed by the Asian Working Group for Sarcopenia 2019 and other studies^21,22^.

**Table 2:** Phenotypic and etiologic criteria for the diagnosis of malnutrition by GLIM. *Source*: Cederholm et al.^9^.

GLIM-DCM	Thresholds	Alternative measurements
Phenotypic criterion		
(i) Nonvolitional weight loss	> 5% within past 6 months or > 10% beyond 6 months	–
(ii) Low BMI	BMI < 18.5 kg/m^2^ for age < 70 years old and < 20 kg/m^2^ for age ≥ 70 years old	–
(iii) Reduced muscle mass	SMI < 7.0 kg/m^2^ for males, < 5.7 kg/m^2^ for females (using dual-energy absorptiometry, bioelectrical impedance, ultrasound, computed tomography or magnetic resonance imaging)	Decreased calf-circumference: Males < 34 cm and females < 33cm^21^ Decreased mid-upper arm circumference: Males < 23.0 cm and females < 22.0cm^22^ Supportive measures: Low handgrip strength: Males < 28 kg and females < 18kg^21^
Etiologic criterion		
(i) Reduced food intake	< 50% of energy requirement for > 1 week, or any reduction for > 2 weeks, or any chronic gastrointestinal condition that adversely impacts food assimilation or absorption (e.g. short bowel syndrome, pancreatic insufficiency, gastroparesis, and intestinal obstructions)	Supportive indicators: Nausea, vomiting, diarrhoea, constipation, abdominal pain, and dysphagia
(ii) Inflammatory conditions	Acute disease/injury (e.g. major infections, burns, trauma or closed head injury) or chronic disease related (e.g. chronic obstructive pulmonary disease, cancer, congestive heart failure, chronic renal disease, liver disease, rheumatoid arthritis, etc.)	–

*BMI* Body mass index, *SMI* Skeletal muscle mass.

### Body mass index

Body weight was measured using an electronic scale (Seca 803, USA) to the nearest 0.1 kg, and body height was measured using a portable stadiometer (Seca 206, USA) to the nearest 0.1 cm. Two consecutive measurements were taken, and the average of the two was used. The BMI of the participants was calculated using the Quetelet’s index: BMI = body weight (kg)/ height (m^2^). Low BMI was defined as having BMI < 18.5 kg/m^2^ for age < 70 years old and < 20 kg/m^2^ for age ≥ 70 years old^9^.

### Unintentional weight loss

The participants were initially asked for their usual weight. The current weight was obtained through a weighing scale and compared to their usual weight. Unintentional weight loss was defined as weight loss of > 5% within past 6 months or > 10% beyond 6 months^9^.

### Reduced muscle mass

A trained clinical dietitian performed physical examinations to identify reduced muscle mass by measuring MUAC and CC. MUAC was measured on the non-paralytic arm using a measuring tape from the midpoint on the triceps between the acromion and the olecranon process. Similarly, CC was measured in a sitting or supine position with the non-paretic knee bent at 90 degrees. Additionally, handgrip strength was measured using the Takei Digital Grip Strength Dynamometer (Model T.K.K.5401) following the user manual's^23^. The TKK dynamometer has good reliability and validity^24^. The participants were excluded from the assessment if there is a visible limitation on tested hands (missing arm, hand, thumb, or finger, wearing a cast on wrist or hand, or most of the hand covered by bandages), had surgery on their hands or wrists in the past three months, participants refuse to take the measurement or unable to obtain a proper testing form. The Asia Working Group for Sarcopenia proposed low handgrip strength as males < 28 kg and females < 18 kg and decreased calf-circumference as males < 34 cm and females < 33cm^21^.

### Adequacy of dietary energy intake

The daily means of energy intake of the participants were assessed using the 7-day Dietary History Questionnaire by a trained clinical dietitian through face-to-face interviews. Dietary analysis was conducted using the Nutritionist Pro™ Nutrition Analysis software. In order to determine whether patients achieved their individual energy needs, their basal metabolic rate (BMR) for participants aged 19–60 years old was estimated using the Malaysian sex-specific equations based on age and body weight^25^. Meanwhile, for elderly participants aged more than 60 years old, the Schofield sex- and age-specific equations based on body height were used^26^. The equations used to estimate BMR are shown in Table 3. Participants' weekly physical activity (PA) levels were assessed by the short version of the International Physical Activity Questionnaire (IPAQ). Based on the metabolic equivalent of task (MET), the physical activity level (PAL) value of each participant was classified as: 1.4 (low PA), 1.6 (moderate PA) and 2.0 (vigorous PA). Kawakawi et al. (2015) demonstrated that the resting energy expenditure of uncomplicated non-surgical intervention stroke patients was increased during the subacute phase and then gradually declined in the chronic phase and restored to its prior state. The study suggested a stress factor of 1.1–1.2 during the acute phase and 1.0–1.1 during the chronic phase of stroke^27^. A higher stress factor was used if the participant was underweight (BMI < 18.5 kg/m^2^ for < 65 years old and BMI < 22 kg/m^2^ for ≥ 65 years old) during the time of the survey^28^. Energy requirements were calculated by multiplying the individual BMR value by the PAL value and stress factor. The percentage of energy intake adequacy was calculated by dividing the energy intake with the estimated energy requirement. Low or suboptimal energy intake was defined as having less than 50% of the estimated energy requirement for more than one week or any reduction (< 75%) for more than two weeks^9^.

**Table 3:** Basal metabolic rate (BMR) equations.

Age group	Males	Females	References
19–29 years	0.0550 W + 2.480	0.0535 W + 1.994	Ismail et al.^25^
30–60 years	0.0432 W + 3.112	0.0539 W + 2.147	Ismail et al.^25^
> 60 years	0.049 W + 2.459	0.038 W + 2.755	Schofield^26^

BMR is expressed in MJ/day, W = body weight in kg.

### Inflammatory conditions

Participants’ underlying medical conditions were charted from the latest medical records. Any recent acute disease/injury such as major infections, burns, trauma, and closed head injury are indicators of severe acute inflammation^9^. Meanwhile, participants were identified as having mild to moderate inflammation if presented with chronic diseases such as chronic obstructive pulmonary disease, cancer, congestive heart failure, chronic renal disease, liver disease, and rheumatoid arthritis. However, this study did not collect proxy of inflammation such as serum C-reactive protein, albumin, or pre-albumin since all the stroke participants were not in their acute phase of stroke.

Additionally, the severity of malnutrition was further classified into either moderate malnutrition or severe malnutrition based on either one of the phenotypic criterion shown in Table 4^9^.

**Table 4:** Thresholds for severity grading of malnutrition according to GLIM-DCM. *Source*: Cederholm et al.^9^.

Severity	Weight loss	Low BMI	Reduced muscle mass
Stage 1/Moderate malnutrition	5–10% within the past 6 months Or 10–20% beyond 6 months	< 18.5 kg/m^2^ for age < 70 years old and < 20 kg/m^2^ for age ≥ 70 years old	Mild to moderate deficits: MUAC < 23 cm for males and < 22 cm for females^22^ CC Males: < 34 cm, Females: < 33cm^29^
Stage 2/Severe malnutrition	> 10% within the past 6 months Or > 20% beyond 6 months	< 16.5 kg/m^2^ for age < 70 years old and < 18.5 kg/m^2^ for age ≥ 70 years old	Severe deficits: MUAC < 20 cm for males and < 19 cm for females^22^ CC < 32 cm for males, < 31 cm for females

*MUAC* Mid-upper arm circumference, *CC* Calf circumference.

### Health-related quality of life

Participants' health-related quality of life was assessed using the EuroQoL-5 Dimensions- 5 Levels questionnaire. The Malay version of the questionnaire has been validated in the Malaysian population^30^. The participants were asked to rate their health today in five dimensions of health: mobility, self-care, usual activities, pain/discomfort, and anxiety/depression, based on a five-point scale (1–5), indicating increasing severity of problems. An EQ-5D summary index was computed based on a formula that attaches values (weights) to each of the levels in each dimension, with reference to the Malaysian EQ-5D-5L value set published by Shafie et al.^30^.

### Statistical analysis

All statistical analyses were performed using the IBM SPSS version 25.0 for Windows. Chi-squared or Fisher’s test was used to compare differences in the categorical variables between two groups. Meanwhile, Student’s *t*-test or Mann–Whitney U-test was used for continuous variables. Analysis of sensitivity, specificity, positive predictive value (PPV) tests, negative predictive value (NPV) tests, the area under the curve (AUC) and correlation coefficients were conducted to evaluate the concurrent validity of the different malnutrition screening tools against malnutrition GLIM-DCM in stroke patients. Participants were classified into either elderly (≥ 65 years old) or non-elderly (< 65 years old) group. The rating of the validation results (good, fair or poor) for the malnutrition screening tools was given based on the cut-off values proposed by the MaNuEL Consortium (2018)^8^. A screening tool was classified as “good” if both sensitivity and specificity were > 80% and AUC was > 0.8; “fair” if sensitivity or specificity are < 80% but both were > 50% and AUC was in the range of 0.6–0.8; and “poor” if sensitivity or specificity was < 50%, and AUC was < 0.6^8^. Besides, correlation coefficient between different malnutrition screening tools with BMI, MUAC, CC, HGS, dietary energy intake, and EQ-5D summary index were computed using the Spearman Rank test.

### Ethics approval and consent to participate

The Medical Research and Ethics Committee in Ministry of Health, Malaysia [NMRR-19-4024-47231 (IIR)] and UniSZA Human Research Ethics Committee [UniSZA/UHREC/2019/102] approved the study protocol. Written informed consent was obtained from cognitively intact elderly stroke individuals or their proxy if they were having severe aphasia, hearing, vision or cognitive issues.

## Results

### Respondents' characteristics

This study screened 448 respondents from three hospitals during the survey; however, 124 respondents were excluded based on exclusion criteria, and 20 refused to participate. Hence 304 respondents were recruited. More than half of the respondents were males, nonelderly, of Malay ethnicity, had married, were not working, and had attained secondary education levels (Table 5). The majority of them had a first-ever stroke, of ischaemic types, with the most recent stroke duration of more than six months, and had at least two comorbidities and above. The prevalence of malnutrition risk varied according to different screening tools, with the highest prevalence observed in MNA-SF (51.3%), followed by MRST-H (33.6%), MUST (30.6%), MST (24.7%), and NRS-2002 (22.4%).

**Table 5:** Comparison of sociodemographic, clinical, and malnutrition status between elderly and non-elderly groups.

	Total (n = 304)	Non-elderly (n = 212)	Elderly (n = 92)	*P*-value^a^
Age (mean ± SD)	58.34 ± 10.71	53.19 ± 8.22	70.18 ± 4.66	< 0.001^b^
Sex				0.615
Female	134 (44.1)	91 (42.9)	43 (46.7)	
Male	170 (55.9)	121 (57.1)	49 (53.3)	
Race				0.725
Malay	259 (85.2)	182 (85.8)	77 (83.7)	
Chinese and Indian	45 (14.8)	30 (14.2)	15 (16.3)	
Marital status				0.089
Married	225 (74.0)	163 (76.9)	62 (67.4)	
Single/widowed/divorced	79(26.0)	49 (23.1)	30 (32.6)	
Working status				< 0.001
Working	49 (16.1)	45 (21.2)	4 (4.3)	
Not working	255 (83.9)	167 (78.8)	88 (95.7)	
Education levels				< 0.001
Primary and below	90 (29.6)	40 (18.9)	50 (54.3)	
Secondary and above	214 (70.4)	172 (81.1)	42 (45.7)	
Episodes of strokes				0.238
First-ever	254 (83.6)	181 (85.4)	73 (79.3)	
Recurrent	50 (16.4)	31 (14.6)	19 (20.7)	
Types of strokes				< 0.001
Ischaemic	215 (70.7)	137 (64.6)	78 (84.8)	
Haemorrhagic	60 (19.7)	55 (25.9)	5 (5.4)	
Unspecified	29 (9.5)	20 (9.4)	9 (9.8)	
Age of stroke onset (mean ± SD)	55.84 ± 11.07	50.59 ± 8.48	67.92 ± 5.53	< 0.001^b^
Duration of stroke (months)				0.079
0–5	135 (44.4)	87 (41.0)	48 (52.2)	
≥ 6	169 (55.6)	125 (59.0)	44 (47.8)	
Number of comorbidities (mean ± SD)	2.75 ± 1.01	2.63 ± 0.98	3.02 ± 1.05	0.002^b^
At risk of malnutrition				
MRST-H	102 (33.6)	70 (33.0)	32 (34.8)	0.792
MUST	93 (30.6)	65 (30.7)	28 (30.4)	1
MST	75 (24.7)	54 (25.5)	21 (22.8)	0.666
MNA-SF	156 (51.3)	103 (48.6)	53 (57.6)	0.17
NRS-2002	68 (22.4)	38 (17.9)	30 (32.6)	0.007
Malnutrition diagnosis^c^				0.115
No	227 (74.7)	164 (77.4)	63 (68.5)	
Yes	77 (25.3)	48 (22.6)	29 (31.5)	
Severity of malnutrition^c^				0.617
Moderate	25 (32.5)	17 (35.4)	8 (27.6)	
Severe	52 (67.5)	31 (64.6)	21 (72.4)	

*MRST-H* Malnutrition Risk Screening Tool-Hospital, *MNA-SF* Mini Nutritional Assessment-Short Form, *MST* Malnutrition Screening Tool, *MUST* Malnutrition Universal Screening, *NRS-2002* Nutritional Risk Screening, *SD* Standard deviation.

^a^Chi-square test for independence

^b^Independent t-test; Significance level at *P* < 0.05.

^c^Malnutrition diagnosis and severity was defined by the diagnostic criteria for malnutrition proposed by the Global Leadership Initiative on Malnutrition (GLIM-DCM)^9^.

Meanwhile, 25.3% of the stroke participants were diagnosed as malnourished according to the GLIM criteria, with 67.5% of them were severely malnourished. Compared to the non-elderly group, the elderly group was much older, had stroke onset at an older age, and had a higher number of comorbidities. Additionally, a significantly higher proportion of them was from poorer socioeconomic status (not working and lower education levels) and had ischaemic types of strokes than their counterparts. Despite this, the prevalence of malnutrition risk was found to be similar between the two groups across different malnutrition screening tools except for the NRS-2002. The elderly group had significantly higher prevalence of malnutrition risk as compared with the non-elderly group when screened with NRS-2002.

Table 6 compares the sensitivity, specificity, positive predictive value, negative predictive value, and the area under the curve for different screening tools with GLIM-DCM between elderly and non-elderly stroke survivors. It was found that the MRST-H and MUST had good sensitivity and specificity (> 80%) in predicting malnutrition using the GLIM criteria in both age groups. In contrast, MST and MNA-SF had fair validity; meanwhile NRS-2002 had poor to fair validity against GLIM-DCM. MST had fair sensitivity (62.1–70.8%) and good specificity (87.8–95.2%); MNA-SF had good sensitivity (89.7–93.8%) but fair specificity (57.1–64.6%), and NRS-2002 had poor to fair sensitivity (47.9–69.0%) but good specificity (84.1–90.9%) in both age groups. The PPV was highest in MST and MUST (63.0–85.7%) but lowest in MNA-SF (43.7–49.0%). In general, the PPV values were higher in the elderly group as compared to the nonelderly group. High NPV (84.5–97.2%) were found in all screening tools in both age groups. Except for MST (0.793 in elderly group and 0.852 in non-elderly group), other screening tools had excellent AUC values in both age groups (> 0.8).

**Table 6:** Comparison of sensitivity, specificity, positive predictive value, negative predictive value, and the area under the curve for different screening tools against GLIM-DCM.

Screening tool	Variable	Non-elderly (n = 212)	Rating	Elderly (n = 92)	Rating
MRST-H	Sensitivity	91.7%	Good	81.5%	Good
	Specificity	84.1%		84.6%	
	Positive predictive value	62.9%		68.8%	
	Negative predictive value	97.2%		91.7%	
	AUC	0.901		0.891	
MUST	Sensitivity	87.5%	Good	82.8%	Good
	Specificity	86.0%		93.7%	
	Positive predictive value	64.6%		85.7%	
	Negative predictive value	95.9%		92.2%	
	AUC	0.884		0.890	
MST	Sensitivity	70.8%	Fair	62.1%	Fair
	Specificity	87.8%		95.2%	
	Positive predictive value	63.0%		85.7%	
	Negative predictive value	91.1%		84.5%	
	AUC	0.852		0.793	
MNA-SF	Sensitivity	93.8%	Fair	89.7%	Fair
	Specificity	64.6%		57.1%	
	Positive predictive value	43.7%		49.0%	
	Negative predictive value	97.2%		92.3%	
	AUC	0.859		0.852	
NRS-2002	Sensitivity	47.9%	Poor	69.0%	Fair
	Specificity	90.9%		84.1%	
	Positive predictive value	60.5%		66.7%	
	Negative predictive value	85.6%		85.5%	
	AUC	0.834		0.834	

*MRST-H* Malnutrition Risk Screening Tool-Hospital, *MNA-SF* Mini Nutritional Assessment-Short Form, *MST* Malnutrition Screening Tool, *MUST* Malnutrition Universal Screening, *NRS-2002* Nutritional Risk Screening, *AUC* Area under the curve, *GLIM-DCM* Diagnosis criteria for malnutrition by the Global Leadership Initiative on Malnutrition^9^.

Tables 7 and 8 display the correlations of different malnutrition screening tools with anthropometric, dietary and health-related quality of life among the non-elderly and elderly groups, respectively. Most of the screening tools were significantly correlated with BMI, MUAC, CC, dietary energy intake, HGS and EQ5D index, with better correlations in the elderly group. However, only MRST-H and NRS-2002 were significantly correlated with all parameters regardless of the age group. The MRST-H demonstrated very weak to weak correlations and weak to moderate correlations with these parameters in the nonelderly and elderly group, respectively. Meanwhile, NRS-2002 showed very weak to moderate correlation with all parameters in non-elderly group but weak to moderate correlation in the elderly group. HGS was not significantly correlated with MUST in the non-elderly group. Additionally, HGS did not significantly correlated with MUST, MST and MNA-SF in the elderly group. Among different screening tools, it was found that the correlation between EQ5D index was the highest in MNA-SF (0.419 in non-elderly and 0.523 in the elderly group).

**Table 7:** Correlations of different malnutrition screening tools with anthropometric, dietary and health-related quality of life in non-elderly group.

Parameters	Screening tools				
	MRST-H	MUST	MST	MNA-SF	NRS-2002
Body mass index (kg/m^2^)	− 0.287^a^	− 0.416^a^	− 0.282^a^	0.394^a^	− 0.379^a^
Mid-upper arm circumference (cm)	− 0.212^b^	− 0.343^a^	− 0.231^b^	0.325^a^	− 0.324^a^
Calf circumference (cm)	− 0.288^a^	− 0.387^a^	− 0.298^a^	0.406^a^	− 0.401^a^
Handgrip strength (kg)	− 0.149^c^	− 0.107	− 0.139^c^	0.231^a^	− 0.190^b^
Energy intake (kcal/day)	− 0.313^a^	− 0.382^a^	− 0.341^a^	0.346^a^	− 0.366^a^
EQ5D index	− 0.185^b^	− 0.184^b^	− 0.191^b^	0.419^a^	− 0.194^b^

^a^*P* < 0.001; ^b^*P* < 0.01; ^c^*P* < 0.05; Spearman’s rank correlation coefficient.

**Table 8:** Correlations of different malnutrition screening tools with anthropometric, dietary and health-related quality of life in elderly group.

Parameters	Screening tools				
	MRST-H	MUST	MST	MNA-SF	NRS-2002
Body mass index (kg/m^2^)	− 0.429^a^	− 0.547^a^	− 0.264^c^	0.539^a^	− 0.459^a^
Mid-upper arm circumference (cm)	− 0.467^a^	− 0.467^a^	− 0.236^c^	0.448^a^	− 0.403^a^
Calf circumference (cm)	− 0.489^a^	− 0.433^a^	− 0.235^c^	0.479^a^	− 0.433^a^
Handgrip strength (kg)	− 0.270^b^	− 0.186	− 0.184	0.147	− 0.283^b^
Energy intake (kcal/day)	− 0.498^a^	− 0.472^a^	− 0.410^a^	0.436^a^	− 0.476^a^
EQ5D index	− 0.442^a^	− 0.301^b^	− 0.433^a^	0.523^a^	− 0.352^b^

^a^*P* < 0.001; ^b^*P* < 0.01; ^c^*P* < 0.05; Spearman’s rank correlation coefficient.

## Discussion

This study shows that both MRST-H and MUST exhibited high sensitivity, high specificity, strong positive and negative predictive values, and good AUC with the GLIM-DCM in both elderly and non-elderly stroke patients. Meanwhile, MST, MNA-SF and NRS-2002 showed poor to fair validity with the reference standard. Only MRST-H and NRS-2002 scores were significantly correlated with all nutritional parameters (e.g., BMI, MUAC, CC and HGS), dietary energy intakes, and health-related quality of life in both age groups.

According to the GLIM-DCM, the overall prevalence of malnutrition diagnosis was 25.3%, which was in line with previous findings among Japanese stroke patients in the acute phase (28.7%)^31^. Shimizu et al., however, showed a higher prevalence at 64.8% among older stroke patients (mean age ± SD = 78.9 ± 7.7 years) with oropharyngeal dysphagia. Similarly, studies in Japan and Australia found a higher prevalence of malnutrition at 46.0–52.0% among older adults admitted to rehabilitation wards (not specific to stroke cases)^32,33^. The discrepancy in results may be due to differences in patient characteristics (younger versus older adults) and assessment time (chronic versus subacute phase). However, our study had found that two-thirds of these malnourished patients had severe malnutrition, raising the possibility of delayed nutritional treatment due to inadequate nutritional screening or worsening nutritional status during the rehabilitation phase^34^. Sremanakova et al., in a clinical audit, showed that malnutrition risk screening (using MUST) was absent in one-third of the stroke patients admitted to the ward in the UK. Additionally, resources for nutritional screening and care may be more limited in the rehabilitation phase than in the acute phase. Early identification of malnutrition risk using a valid malnutrition screening tool allowed better nutritional care and lower malnutrition incidence in the clinical setting^35^. Malnutrition among stroke survivors is undesirable as it is associated with poor functional recovery and clinical outcomes (mortality, length of hospital stay and hospitalization cost)^36,37^. Thus, early identification of malnutrition with validated malnutrition screening tools is highly recommended.

The prevalence of malnutrition risk as screened using different screening tools ranged between 22.4 and 51.3% in this study. Our prevalence was higher than those reported at 18.1–38.5% in a Vietnam study in acute care which included adults patients from medical and surgical wards but excluded those under rehabilitation care^38^. When choosing the appropriate malnutrition screening tools, a clinician must ensure that the tool is validated, with good sensitivity and specificity to the targeted population. For instance, NRS-2002 was developed mainly for adults in the acute setting; meanwhile, MRST-H and MNA-SF were specifically designed for the elderly population in acute and community settings^8,15,16^. This might explain the discrepancy in the prevalence of malnutrition risk detected across different tools. Unsurprisingly, NRS-2002 was the least preferable screening tool in the rehabilitation setting, given its poor to fair sensitivity and specificity against GLIM-DCM. This is probably because one of the important criteria in the NRS-2002, namely severity of disease in the acute phase, was less applicable among community-dwelling stroke patients.

In contrast, the prevalence of malnutrition risk was the highest when screened by the MNA-SF. However, we noticed that MNA-SF has the lowest specificity as compared with other screening tools, thus might increase the chances of inappropriate referrals for further assessment for those who are not truly malnourished. Specificity is particularly important to consider in service provision and staffing resources^39^. Similarly, Nishioka et al. suggested that MNA-SF may potentially overestimate the prevalence of malnutrition among elderly stroke patients in the rehabilitation phase (due to its low specificity), with a prevalence of 91–99% being reported in the Asian population^40,41^. This was because it was difficult to differentiate the characteristics of malnutrition with stroke-related impairments, including reduced mobility caused by hemiparesis, communication problems due to aphasia, and unintentional muscle atrophy following stroke, causing sub scoring of many items in the MNA-SF^42^. As compared to our study, Marshall et al.^17^ and Nishioka et al. (2019) reported higher sensitivity (100%) but lower specificity (1.7–22.6%) for MNA-SF among patients in rehabilitation. Differences in the methodology might explain the discrepancy in the results. First, we excluded unfit patients for anthropometric measurements, thus providing better accuracy for the BMI assessment. Second, the reference standard used, namely GLIM-DCM, ESPEN-DCM, and ICD-10-DCM, would result in differences in the malnutrition prevalence. The ESPEN-DCM has similar phenotypic criteria to the GLIM-DCM (weight loss, low BMI, and low muscle mass); however, it does not include the aetiology of malnutrition (inflammatory conditions or reduced food intake)^6^. Meanwhile, although the ICD-10-DCM includes an assessment of BMI, weight loss, food intake and muscle loss, yet details regarding a timeframe for weight loss or the degree of reduced food intake are absent^43^. Shimizu et al. showed that the GLIM-DCM, which includes the aetiology of malnutrition as a component, could identify malnutrition more frequently than the ESPEN-DCM in geriatric rehabilitation care units^32^.

On the other hand, our study showed that the accuracy of the MST in reference to GLIM-DCM was fair regardless of the age group. This is in line with previous data, which reported fair validity of MST when validated against GLIM (56.7% sensitivity, 69.0% specificity and AUC 0.63) among geriatric rehabilitation patients in Australia^33^. Similarly, other Australian studies reported fair validity of MST when validated against ICD-10-DCM and SGA among patients in the rehabilitation facilities (including neurological/stroke cases): sensitivity 72.2–80.8% and 67.7–83.8% specificity^17,44^. Despite its fair validity, MST is the quickest and easiest to administer screening tool since only two items are involved, and no anthropometric measurements are needed. Further evidence from Asian countries, however, should be obtained before concluding the usefulness of MST in the rehabilitation/stroke patient.

We observed that MRST-H and MUST had high sensitivity and specificity against GLIM-DCM in both age groups, which seemed to be an appropriate screening tool for the stroke population. It is unlikely to compare our findings with previous data since MUST and MRST-H have not been validated among stroke survivors or rehabilitation setting. However, these screening tools have been validated among adults in either community or residential care, with fair to good validity^8,15,45^. Tan et al.^15^ conducted concurrent validation of the MRST-H against Subjective Global Assessment (SGA) in hospitalized and outpatient visiting elderly. The sensitivity and specificity values of the MRST-H were reported at 67% and 90%, respectively. In contrast, Tran et al.^38^ conducted validation of different screening tools, namely MUST, MST, NRS-2002 and MNA-SF, among adults admitted to medical and surgical wards. The study, however, found that NRS-2002 (sensitivity 74.6%; specificity 80.6%) and MUST (sensitivity 63.4%; specificity 85.2%) showed fair validity, meanwhile MST (sensitivity 41.8%; specificity 82.0%) and MNA-SF (sensitivity 35.0%; specificity 95.8%) had poor validity when compared to the reference standard SGA and BMI.

Apart from concurrent validity and reliability, the ability of the malnutrition screening tool to predict specified clinical outcomes is also important. Studies in the UK have demonstrated that MUST is an independent predictor of mortality, length of stay and hospitalization costs at six months after stroke^34,36^. Similarly, Zhang et al. showed that MUST scores were significantly associated with the modified Rankin Scale at 12 months post-discharge from a stroke event in China^46^. Meanwhile, Marshall et al.^17^ showed that neither the MST nor MNA-SF could predict rehospitalization, institutionalization, or discharge location among older adults in rehabilitation. The validity of MRST-H in predicting clinical outcomes among stroke survivors in Malaysia, however, was not being examined in this study and thus need further investigation.

Surprisingly, we did not observe a significant difference in the prevalence and severity of malnutrition between the elderly and non-elderly groups across most of the screening tools except for the NRS-2002. This is probably because only NRS-2002 considered age ≥ 70 years as an additional risk of malnutrition in the screening tools. The insignificance results might also be explained by differences in the stroke subtypes where a higher prevalence of haemorrhagic stroke was observed in the non-elderly patients. Some studies have shown that as compared to ischaemic stroke, haemorrhagic stroke is more likely to be associated with high malnutrition risk^31,36^ and an increased energy need, particularly for those who underwent surgical intervention^47^. The relationship between stroke subtypes and nutritional status remained insufficiently investigated and warrants further study. Additionally, the prevalence of stroke-related impairments was common among the stroke patients regardless of their age in this study. Neurological deficits post-stroke, such as dysphagia, hemiparesis, cognitive impairment, visual and speech deficits, have hindered their ability to consume adequate nutrition^48^.

All of the screening tools were significantly associated with BMI, MUAC, CC, energy intake, and health-related quality of life. However, only MRST-H and NRS-2002 were significantly associated with HGS in both age groups. This is probably because the MRST-H had included muscle mass (MUAC and CC); meanwhile, NRS-2002 considered age as an additional malnutrition risk as part of the screening items. Although HGS has been treated as one of the nutritional markers indicating early nutritional deprivation, HGS is confounded by other factors such as the presence of hemiparesis in stroke, stroke recovery process and ageing. Meanwhile, the correlation between MNA-SF with EQ5D-index was the highest when compared to other screening tools. This is probably because MNA-SF has included mobility and depression items, which are part of the parameters in computing the EQ5D summary index.

Apart from accuracy in predicting malnutrition, clinicians should be aware of the limitations and advantages of each nutrition screening tool. First, unlike MNA, MUST and NRS-2002, MST and MRST-H do not require measurement of BMI. Measuring accurate weight and height is challenging, particularly among stroke patients with hemiparesis, and thus it is often not routinely conducted in the clinical setting. Our study had excluded almost one quarter of the eligible participants since they could not stand properly. Second, only MRST-H and MNA had considered the use of CC (compulsory measurement in MRST-H but optional for MNA-SF). CC is a good predictor of muscle wasting and correlates well with a functional capacity which is important in stroke recovery^40^. Unlike the Geriatric Nutritional Risk Index, all these screening tools do not require any laboratory data; thus, they are more cost-effective and are considered non-invasive tools. Fourth, to reduce the screening burden on patients and clinicians, most screening tools have relatively few items to administer, with the lowest items found in MST (2 items) and the highest seen in MNA-SF (7 items). Last but not least, MUST, MNA-SF and NRS-2002 considered the acute disease effects in its criteria, and only MNA-SF included psychological stress factor. Further investigation on the feasibility and predictive validity of these malnutrition screening tools is required in future studies.

This was probably one of a few multicentre studies which examined the validity of different malnutrition screening tools with the latest GLIM-DCM in an Asian rehabilitation setting. Therefore, we believed that the study findings will contribute important information regarding importance of choosing the appropriate malnutrition screening tools in stroke rehabilitation setting. Despite this, since the study was confined to two states of Malaysia; thus, the generalisation of the findings to the whole Malaysian population was limited.

## Conclusion

We can conclude that the MUST and MRST-H showed good concurrent validity with GLIM-DCM and can be considered as appropriate malnutrition screening tool in discriminating malnutrition among stroke individuals attending rehabilitation centre in Malaysia regardless of their age groups. The prevalence of malnutrition among stroke individuals in rehabilitation phase was high at 25.3%. Therefore, screening and assessment for malnutrition should be incorporated into the standard stroke care to allow early treatment of malnutrition, improve patient and health outcomes and prevent complications.

## Data Availability

The data that support the findings of this study are available from the corresponding author. However, restrictions apply to the availability of these data and permission from the Ministry of Health Malaysia is required.
